# Leveraging structural and 2D-QSAR to investigate the role of functional group substitutions, conserved surface residues and desolvation in triggering the small molecule-induced dimerization of hPD-L1

**DOI:** 10.1186/s13065-022-00842-w

**Published:** 2022-06-27

**Authors:** Marawan Ahmed, Aravindhan Ganesan, Khaled Barakat

**Affiliations:** 1 Faculty of Pharmacy and Pharmaceutical Sciences, University of Alberta, Edmonton, AB Canada; 2 ArGan’s Lab, School of Pharmacy, University of Waterloo, Kitchener, ON Canada; 3 Li Ka Shing Institute of Virology, University of Alberta, Edmonton, AB Canada

**Keywords:** Immune-checkpoints, Programmed cell death ligand 1 (PD-L1), Small molecule drugs, Computational solvent mapping, Free-Wilson, R-group decomposition

## Abstract

Small molecules are rising as a new generation of immune checkpoints’ inhibitors, with compounds targeting the human Programmed death-ligand 1 (hPD-L1) protein are pioneering this area of research. Promising examples include the recently disclosed compounds from Bristol-Myers-Squibb (BMS). These molecules bind specifically to hPD-L1 through a unique mode of action. They induce dimerization between two hPD-L1 monomers through the hPD-1 binding interface in each monomer, thereby inhibiting the PD-1/PD-L1 axis. While the recently reported crystal structures of such small molecules bound to hPD-L1 reveal valuable insights regarding their molecular interactions, there is still limited information about the dynamics driving this unusual complex formation. The current study provides an in-depth computational structural analysis to study the interactions of five small molecule compounds in complex with hPD-L1. By employing a combination of molecular dynamic simulations, binding energy calculations and computational solvent mapping techniques, our analyses quantified the dynamic roles of different hydrophilic and lipophilic residues at the surface of hPD-L1 in mediating these interactions. Furthermore, ligand-based analyses, including Free-Wilson 2D-QSAR was conducted to quantify the impact of R-group substitutions at different sites of the phenoxy-methyl biphenyl core. Our results emphasize the importance of a terminal phenyl ring that must be present in any hPD-L1 small molecule inhibitor. This phenyl moiety overlaps with a very unfavorable hydration site, which can explain the ability of such small molecules to trigger hPD-L1 dimerization.

**Supplementary Information:**

The online version contains supplementary material available at 10.1186/s13065-022-00842-w.

## Introduction

Immunotherapy is emerging as a transformative paradigm in the treatment of cancer. This new paradigm relies on releasing the brakes of the immune system, allowing it to recognize and destroy cancer cells [1]. One of these brakes is the Programmed cell death protein 1 (PD-1), where blocking this receptor and its ligand, PD-L1 with monoclonal antibodies (MABs) has revolutionized cancer treatment over the last few years [2]. However, despite their outstanding success, these MABs still have numerous disadvantages, including cost and side-effects [3–6]. Second-generation MAB checkpoint inhibitors target either the receptors or ligands involved in non-PD-1 pathways. Promising examples target the cytotoxic T lymphocyte–associated antigen 4 (CTLA-4) receptor (*e.g.* Ipilimumab [7]). However, with all of these MABs a spectrum of severe immune-related adverse events (irAE) started to emerge [3], which include rash, diarrhea, colitis and hepatotoxicity. Although combining multiple immune checkpoints inhibitors has enhanced the overall efficacy [8], their side-effects are still of concern. Furthermore, these MABs are very expensive to manufacture and administer, making them financially inaccessible to many patients. For example, the treatment cost per quality-adjusted life year for a patient with metastatic melanoma using anti-CTLA-4 MAB exceeds $500,000 (USD) [9, 10]. In this context, the introduction of small molecule immune checkpoints’ inhibitors represents a third-generation wave of alternatives to these MABs. These small molecules have the potential of enabling the efficient treatment of many existing cancer types at a reasonable cost and manageable side effects.

Of all immune checkpoints, PD-L1 seems to be the most promising target for small molecule inhibitors [11–13]. PD-L1 (also known as CD274 or B7-H1) is constitutively expressed on antigen presenting cells (APCs) and on the surface of non-hematopoietic organs such as lung and heart. PD-L1 and its homolog, PD-L2 (37% sequence homology), are the two well-characterized endogenous protein ligands for PD-1 [14–16]. The PD-1/PD-L1 pathway suppresses T-cells activity, up-regulates regulatory T cells (Treg) that are involved in promoting self-tolerance and reduces autoimmunity [17, 18]. PD-L1 was also shown to bind to B7-1, another immune-checkpoint ligand, to deliver an inhibitory signal to the immune system [19]. The overexpression of PD-L1 on the surface of many types of cancer cells attenuates the directed immune response against these cells, leading to a state of immune escape [20–23]. Directed therapy against the PD-L1 ligand is a successful strategy to reactivate the immune system to recognize and kill these cancer cells [24, 25].

Researchers at Bristol-Myers-Squibb (BMS) have pioneered the discovery of small molecule inhibitors against PD-L1. They have recently disclosed several patents describing the chemical structures, synthetic routes and the homogeneous time-resolved fluorescence (HTRF) binding assay data for a number of their compounds. This includes a new class of (2-methyl-3-biphenylyl) methanol derivatives that serve as potent (nano-molar) inhibitors of the human PD-1/PD-L1 interactions [26, 27]. Two follow-up X-ray structure studies have confirmed the binding mode of these small molecules onto a groove formed at the interface between two PD-L1 monomers, at their PD-1 binding faces [28, 29]. Using a combination of classical and accelerated MD simulations, we have recently investigated the nature of this binding site. It was evident from our analysis that this site is a cryptic site that is transiently accessible for a limited period of time [30]. The clinical benefits of many small molecules from this series have been demonstrated in humanized mouse models [31].

**Fig. 1:**
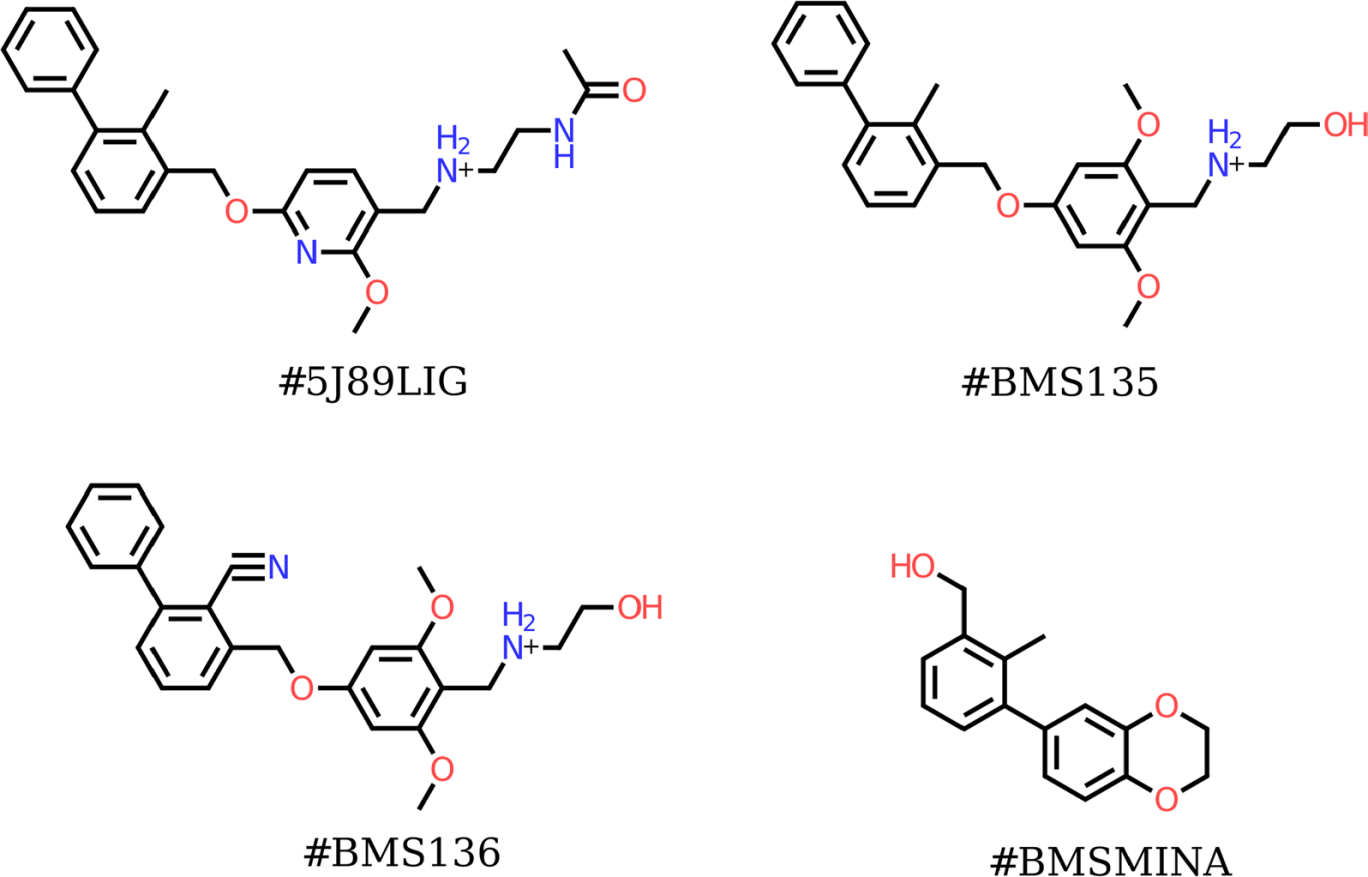
The chemical structures of the molecules under study; #5J89LIG, #BMS135, #BMS136, and BMSMINA

The current study provides a “deep-dive” into the dynamicity and druggability of this PD-L1 cryptic site. Here, we describe and discuss the results obtained from a detailed in silico structural characterization of five small molecule organic compounds targeting the PD-L1 protein. The chemical structures of these molecules are shown in Fig. 1. The potential binding modes of these compounds are discussed below in light of in vitro characterization data obtained by our group as well as additional data reported in the literature [32]. The computational methods used in this study include molecular docking simulations, molecular dynamics simulations, binding free energy calculations and Computational Solvent Mapping through Grid Inhomogeneous Solvation Theory (GIST) and Hydration Site Analysis. Furthermore, ligand-based analyses, including Free-Wilson 2D-QSAR was conducted to quantify the impact of R-group substitutions at different sites of the phenoxy-methyl biphenyl core. A virtual library of potential molecules was built around the core, scored and made ready for the next steps. We hope the findings described in this work can advance the development of more potent PD-L1 inhibitors and be translated to other immune checkpoint receptors.

## Methods

### Preparation of the protein–ligand complexes

The X-ray crystal structure of hPD-L1 dimeric structure co-crystallized with a small molecule compound from a recent BMS patent (PDB ID: 5J89) was used to prepare all protein-small molecules’ complexes described below [28]. The initial PDB structure was prepared using the protein preparation wizard in Schrodinger software [33]. Preparation includes the completion of missing protein residues and heavy atoms, building disulphide bonds, the addition of hydrogen atoms and partial charges, and the proper assignment of the protonation states of titrable amino acid residues at pH 7. Finally, restrained partial minimization of the complex was performed and the restraints were released when the root-mean-square deviation of the heavy atoms reached 0.3 Å. The recent forcefield from Schrodinger, namely OPLS3-FF [34], was used.

The chemical structures of all studied molecules (see Fig. 1) were constructed using Maestro and were prepared using ligprep. Preparation included adding hydrogen atoms, assigning proper protonation states at the corresponding pH (*i.e.* pH = 7) as well as optimizing ligand geometries. Following ligand preparation, molecular docking simulations were performed through the standard precision mode of Glide (Glide SP), and the best scoring pose of each ligand (according to the Glide SP score) was saved for further in silico structural analysis. To validate the docking protocol, the co-crystallized ligand (#5J89LIG) was redocked to the crystal structure. The resulting root mean square deviation (rmsd) value of the top-scoring poses (according to the glide docking score) was found to be < 1 Angstrom. The deviation from the co-crystalized ligand was mainly centered around the linear solvent-exposed structural motif of the compound.

The five compounds selected in this work include a small molecule that was disclosed by BMS and was described in a recent crystal structure with PD-L1 (PDB ID: 5J89) [28]. This compound is referred to as #5J89LIG in this study. The selected compounds also include two reported PD-L1 inhibitors, referred to as #BMS135, #BMS136, used internally by our group as positive controls in our immune-checkpoint program [32]. In addition, we selected a minimal active fragment that was reported in a recent NMR-based study by Skalniak et al. [35], named here as #BMSMINA. This fragment represents a minimal requirement to induce a PD-L1 dimerization as indicated in the H^1^-N^15^ HMQC NMR chemical shift experiments conducted by Skalniak et al. It includes the biphenyl ring system (of the 2-methyl-3-biphenylyl methanol motif) which is mandatory for binding and for triggering the dimerization of two PD-L1 protein monomers. It is important to note that molecules lacking this biphenyl ring system failed to show any activity towards PD-L1 [35].

### Preparation of protein–protein complexes

In addition to studying the interactions of PD-L1 with small molecules, we also studied the interactions of PD-L1 with PD-1 and PD-L1 in the absence of small molecule inhibitors. This was done to understand the molecular mechanism utilized by such inhibitors to induce a new receptor-side dimer formation in PD-L1. Towards this goal, we studied the structures of three additional PD-L1-mediated complexes. This included an X-ray crystal structure of human PD-1/PD-L1 complex (PDB code: 4ZQK) [36, 37]; a physiological PD-L1/PD-L1 dimer (the back-to-back dimer as described in PDB code: 5JDR [38]), and a small molecule induced dimer (face-to-face dimer), after removing the bound small molecule. For the third system, the PDB: 5J89 structure was used as a starting structure, and the small molecule gluing ligand was removed from this complex [28].

### Molecular dynamics simulations

In total, we ran seven classical MD simulations for the above-mentioned systems. That is four MD simulations for PD-L1 with bound ligands and three simulations for PD-L1 in complex with either PD-1 or PD-L1 with no ligands bound. Before starting any MD simulation, each protein–ligand/protein–protein complex was prepared through the tleap module in AMBER16 [39]. For each protein–ligand complex, the atomic partial charges of the ligand were assigned from the resulting electronic wave functions calculated through the semi-empirical AM1 method and fitted to the atomic centers by the restrained electrostatic potential (RESP) procedures in antechamber [40, 41]. Each complex was then immersed in a cubic box of TIP3P water model with a minimum of 12 Å distance between the box boundaries and the closest protein/ligand atoms, neutralized with counter ions and made ready for simulation.

Our explicit solvent MD simulation protocol involved four main stages: (i) energy minimization, (ii) NVT heating, (iii) NPT equilibration and (iv) NPT production simulations. The energy minimization stage was performed over 4 consecutive rounds. The first minimization round was conducted for 5000 minimization steps, for which the first 4000 steps were performed according to the steepest descent (SD) protocol and the last 1000 steps were conducted according to the conjugated gradient (CG) protocol. In the first minimization round, a strong 100 kcal mol^−1^ Å^−2^ harmonic constraint was applied to the protein and ligand atoms. This constraint was gradually released in the following three minimization rounds to 50, 5 and 0 kcal mol^−1^ Å^−2^. Following minimization, each system was gradually heated to 300 K in 50,000 steps using the NVT ensemble, performed with an integration timestep of 1 fs and a weak harmonic restraint of 5 kcal mol^−1^ Å^−2^. The equilibration phase of the simulation was performed over 4 consecutive rounds. In the first round, 25,000 equilibration steps were performed using the NPT ensemble with an integration timestep of 2 fs and a force constant of 1 kcal mol^−1^ Å^−2^ on the heavy atoms of the protein and ligand involved in the complex. These constraints were gradually reduced sequentially as 0.1 kcal mol^−1^ Å^−2^ for 50 ps, 0.01 kcal mol^−1^ Å^−2^ for 50 ps and finally 0 kcal mol^−1^ Å^−2^ for 1 ns. For the production simulation, the 120 ns long MD trajectory was generated by combining 6*20 ns MD simulation trajectories, using an integration timestep of 2 fs. Throughout the simulation, temperature was controlled through the Langevin thermostat [42] and the pressure was kept at 1 bar using the Berendsen barostate [43].

### Binding free energy calculations

All MM-GBSA calculations in this work were carried out using the MMPBSA.py utility in AMBERTools16 [44]. The last 100 ns of the 120 ns long MD simulations trajectory was selected to carry out the MM-GBSA binding free energy calculations as well as other dynamics’ analyses. The MM-GBSA method for estimating binding free energies has been applied in several studies with various levels of success [45–47].

According to the MM-GBSA protocol, implemented in AMBER, the protein–ligand binding affinity (***ΔG***_***bind***_) is calculated as follows:

$$\Delta {\text{G}}_{{{\text{bind}}}} \, = \,\Delta {\text{E}}_{{{\text{MM}}}} \, + \,\Delta {\text{G}}_{{{\text{solv}}}} {-}{\text{T}}\Delta {\text{S}}$$

In the aforementioned equation, G_complex_, G_protein_ and G_ligand_ are the calculated free energies of the complex, the protein and the ligand, respectively, over the considered portion of the MD simulation trajectory. ΔE_MM_ is the gas-phase interaction energy between the protein and the bound ligand, which represents the summation of the electrostatic (ΔE_ELE_) and van der Waals (ΔE_vdW_) energy terms. ΔG_sol_ is the solvation energy term that includes the polar (ΔG_pol_) and the non-polar (ΔG_nonpol_) contributions. In this work, the polar contribution to the solvation free energy was calculated with the Generalized Born (GB) approximation model. The non-polar part was obtained as (ΔG_nonpol_ = γSASA + β). In this equation, SASA is the calculated solvent-accessible surface area; γ and β are constants and were fixed to 0.0072 kcal/mol/Å and 0.0 kcal/mol, respectively. The MMGBSA binding energies were estimated in two different scenarios; (i) the small molecule is a ligand that binds to the receptor which is formed from two loosely bound PDL1 protein monomers; and scenario (ii) the second chain of PD-L1 is a ligand that binds to a receptor formed from a small molecule which is loosely bound to another PD-L1 protein monomer. Unless otherwise specified, all binding energy calculations displayed in the figures or discussed in the text are the mean AMBER-MMGBSA binding energy values collected over 12,488 MD frames, with error bars denoting standard deviations from the mean. 12 MD frames were evenly sampled from the MD trajectory to carry out the normal mode analysis and estimate the entropic changes.

### Computational solvent mapping through grid inhomogeneous solvation theory (GIST) and hydration site analysis (HSA)

All protein–ligand/protein–protein binding reactions are usually preceded by displacing water molecules from the corresponding hydration sites at the interacting surfaces [48, 49]. Given the unique binding mechanism of the reported PD-L1 inhibitors, we were interested in investigating if water has any effect on triggering this unusual binding mode. Towards that, we analyzed the thermodynamic properties of water molecules surrounding the PD-L1 protein, through quantifying their enthalpy and entropy. A deep understanding of these two terms can shed light on the observed binding mechanism and help designing better molecules with enhanced biological activities.

From the plethora of computational techniques available in the literature to investigate the thermodynamic properties of water molecules in biological systems, the Grid Inhomogeneous Solvation Theory (GIST) and Hydration Site Analysis (HSA) stand out. Whereas HSA outputs these thermodynamic properties per hydration site, GIST discretizes these quantities onto three-dimensional grids. GIST was first proposed by Nguyen et al. [50] in a seminal study aimed at understanding the hydration properties of the cucurbit[7]uril receptor. The method has been applied successfully in a number of biomolecular research studies aimed at quantifying the thermodynamic properties of water molecules at various ligand-binding as well as enzyme active sites [51, 52].

From a technical point of view, GIST and HSA accepts the output (MD frames) of a restrained, explicit solvent biomolecular MD simulation and uses these MD frames to calculate localized thermodynamic properties of water at a given hydration site. In GIST, these thermodynamic properties include water densities, enthalpies, entropies, and free energies within discretized three-dimensional rectangular grid boxes (voxels, *k*), usually of a ~ 1 A^3^ volume. The outputs of a GIST and HSA analyses are immense, including water occupancies and several thermodynamic quantities to estimate solute–solute as well as solute–solvent interactions. For a detailed list of full GIST and HSA outputs, please refer to the literature, examples given here [52], here [52] and here [51]. The corresponding AMBER documentation on GIST and HSA analyses (GIST and HSA are implemented in CPPTRAJ) is another valuable source to serve that purpose. A short list of these thermodynamic quantities that we found useful is the following:

**ΔE**_**sw**_: Average absolute solute-water binding energy at *hydration site*.

**ΔE**_**ww**_: ½ Average absolute water-water binding energy at *hydration site*. The one-half factor is added to prevent the double counting.

** − TΔS**_**sw**_**:** single-body (one-water) translational and orientational entropies in, relative to bulk, normalized to the number of waters in the *hydration site*.

In this context, the absolute total enthalpies (**ΔH**) of water molecules (normalized to a single water molecule) can be written as:

$$\Delta {\text{H = }}\Delta {\text{E}}_{{{\text{sw}}}} \, + \,{2}*\left( {\Delta {\text{E}}_{{{\text{ww}}}} - \Delta {\text{E}}_{{{\text{bulk}}}} } \right)$$

Therefore, the free energies of water molecules (normalized to a single water molecule) at can be written as:

$$\Delta {\text{G = }}\Delta {\text{H}}\, - \,{\text{T}}\Delta {\text{S}}_{{{\text{sw}}}}$$

Thus, unfavourable waters (easy to displace, unhappy water) will generally have higher free energies than favourable (hard to displace, happy water) ones [52, 53].

We have also implemented a scoring system to quantify the effect of each site. Multiplying the water occupancies at a given site by the total energies (**ΔE**_**sw**_** + ΔE**_**ww**_) should give an estimate of this effect, provided that the energy of bulk water is considered. In our subsequent analysis, we will refer to this score as the site score (*k*_*ss*_), which could be given by:

$${\text{Kss}}\, = \,{\text{Site}}\,{\text{Occupancy }}\left( {\Delta {\text{E}}_{{{\text{sw}}}} \, + \,\Delta {\text{E}}_{{{\text{ww}}}} {-}{9}.{565}} \right)$$

Where − 9.565 is the TIP3P water model mean energy (E_ww,bulk_) (kcal/mol/water) of a single water molecule, as suggested by a study from Nakano et al. [54]. The lower the ***Kss*** score the harder the displacement of water molecules from this site, and vice versa.

In the current study, we used a single chain of the 5J89 PD-L1 protein structure as an input for a restrained MD simulation. All simulation details, including simulations set-up, minimization and equilibration simulations are similar to what was discussed earlier in this study with the exception that the production simulation was performed for 120 ns (6*20 ns) ns with a 2 kcal mol^−1^ Å^−2^ restrains on protein heavy atoms. Prior to performing the GIST and HSA analyses, all frames of the trajectory were RMS-fitted to the starting structure of the trajectory. This was critical to eliminate the effect of the global translational and rotational motions of the protein atoms. Fitting was performed on the Cartesian coordinates of all protein heavy atoms. We used a grid spacing of 0.5 Å, and the calculations were performed for 60,000 frames spaced at 2 ps interval. GIST and HSA analyses were performed using the CPPTRAJ implementation in AMBER16.

### 2D QSAR modeling: Free Wilson analysis and virtual library enumeration

To further investigate the impact of the different functional group substitutions on the biological activities of PD-L1 inhibitors, we employed 2D QSAR modeling. Specifically, we adopted the Free Wilson analysis (FWA) on the 3-(Phenoxymethyl)biphenyl series. In the FWA, the structural features of the ligands are directly correlated with the observed biological activities. In practice, the chemical structures of a closely related series of bioactive ligands are decomposed into several R-groups, decorating a core scaffold. Once the core scaffold has been determined, each substitution site in each molecule in the series is one-hot encoded (i.e. featurized) according to the presence or absence of a given functional group from the pool of R-groups substitutions in the entire series. Subsequently, an explainable regression model is built to correlate the biological activities (dependent variable) to the structural features (independent variables). The signs of coefficients of this model represent the correlation direction and the values represent the magnitude to which the corresponding functional group modulates the biological activity. Free-Wilson analysis is a gold standard technique in 2D QSAR modeling and has been successfully applied in many studies to understand the effect of functional groups substitutions in several medicinal chemistry campaigns [55–57].

Our analysis started by collecting a representative dataset with corresponding activities. For this purpose, a local copy of the entire BindingDB was downloaded from (https://www.bindingdb.org/bind/chemsearch/marvin/SDFdownload.jsp?all_download=yes) as a single tab-separated file (BindingDB_All_2021m7.tsv.zip). Records with activity flags against the Programmed cell death ligand 1 protein (PD-L1) were selected. Our QSAR analysis was focused only on the original, BMS series that had the 3-(Phenoxymethyl)biphenyl core, therefore, dimerized ligands, ligands that belong to other core scaffolds, or ligands that bear the dioxane-biphenyl core, as well as ligands missing activity or structural information, were not included. Furthermore, the bioactivity values of ligands with multiple activity records were averaged and duplicate records were dropped (a single record for each ligand was kept for analysis). The final dataset contained 403 unique ligands with an IC_50_ values range of 4.55 nM to 14,250.0 nM. The IC_50_ values were converted to the log scale (pIC_50_ = − log(IC_50_)). The dataset was used as the input for building the Free-Wilson-based regression model.

For the Free-Wilson analysis, the 3-(Phenoxymethyl)biphenyl scaffold was considered as a core scaffold for the selected dataset, and seven R groups substitutions sites were determined on this core. Although many novel and potent BMS-like scaffolds have been recently disclosed by several groups that are actively working in the field [58, 59], the scarcity of available datasets prevented us from performing similar analyses for these scaffolds. We are currently working on collecting more data on these scaffolds and the results will be presented in future studies.

The Free-Wilson GitHub repository (https://github.com/PatWalters/Free-Wilson) was used to; (i) decompose the input chemical series to the corresponding, R-groups one-hot encoded vector, (ii) build the ridge regression model of the input molecules; ii) enumerate a virtual library of unexplored substituent combinations to create novel molecules and predict the pIC50 values of these novel molecules using the developed regression model. Functional groups’ coefficients (variable weights) were also generated. For the regression model, the RidgeCV model was used. RidgeCV is a regression model with a built-in Leave-One-Out Cross-Validation protocol to prevent overfitting. For more details about the model, please refer to the corresponding scikit-learn documentation:

https://scikit-learn.org/stable/modules/generated/sklearn.linear_model.RidgeCV.html

## Results and discussion

One of the hallmarks of cancer is the ability of tumors to evade immune responses [60]. CD8^+^ cytotoxic T lymphocytes (CTLs) play a crucial role in eliminating tumor cells. However, the destructive capacity of CTLs is progressively dampened and CTLs become dysfunctional during cancer development. This is mediated by expression of several receptors (*e.g.* PD-1 and CTLA-4) on CTLs, called immune checkpoints. Tumors can attenuate the activity of CTLs by upregulating ligands for these receptors [61] and, hence, blocking these interactions restores exhausted CTL function and reactivates the immune system to recognize and kill tumor cells [62, 63].

In this context, monoclonal antibodies targeting the immune checkpoints’ receptors have revolutionized cancer therapy for the last decade. However, their cost and frequent severe side effects represent a vast barrier against their broad adoption in healthcare systems. Small molecule immune checkpoints inhibitors, hence, offer a practical rectification to this problem, with inhibitors targeting PD-L1 leading the way towards this goal. In order to understand the mode of action of current PD-L1 small molecule inhibitors, with the ultimate goal of translating this knowledge to other immune checkpoints’ targets, we focused on studying the binding of several PD-L1 inhibitors. The chemical structures of these compounds are shown in Fig. 1. We used the co-crystalized compound, namely #5J89LIG (PDB ID: 5J89) as the basis of comparison for all other small molecules. #5J89LIG is an efficient hPD-1/hPD-L1 inhibitor with an IC_50_ value that is given by 18.0 nM (CHEMBL Assay ID: CHEMBL4017391). Compound #BMSMINA has been described in a recent study by Skalniak et al. [35], confirming the importance of the biphenyl ring moiety as the minimal structural element required to elicit a bio-molecular association signal in NMR based binding experiments. The study also showed that a mono-phenyl ring system failed to show any binding activity in the performed 2D-NMR binding experiment.

### Conventional and non-conventional protein–protein interaction (PPI) inhibitors

The observed inhibition mechanism of the hPD-1/hPD-L1 PPI by the molecules reported by BMS is relatively uncommon compared to classical PPI inhibition. The vast majority of “conventional” PPI interaction inhibitors tend to bind to one of the interacting proteins at the binding interface with the other protein, hence, preventing the PPI [64–66]. An example of this approach is navitoclax (ABT263), a small molecule discovered by Abbott laboratories, which disrupts the interactions of the antiapoptotic protein, Bcl-2, with apoptosis-executing proteins (*e.g.* Bad, Bid and Bak) [67]. In addition to this classical PPI inhibition approach, certain examples in the literature show that it is also possible to disrupt a PPI through stabilizing protein–protein complexes. This strategy relies on the fact that proteins are highly dynamical macromolecules that usually exert their biological functions through a tightly controlled cascade of association-dissociation events with their bio-molecular partners [68]. Therefore, an extra stabilization of a physiologically relevant protein–protein complex or the formation of a non-physiological complex can lead to the same overall biological effects resulting from a conventional PPI inhibition. That is disrupting the physiological pathway. Examples of small molecules that achieve PPI inhibition through this protein–protein stabilization approach include dexrazoxane, which stabilizes the closed conformation of the DNA topoisomerase II homodimer [69], and 1EBIO, which stabilizes calmodulin: potassium-channel interactions [70, 71]. In these complexes, the small molecule works as a “bio-molecular glue”, usually by occupying a pocket between the two proteins and preventing them from performing their normal biological functions. PPI inhibition through a protein–protein stabilization mechanism is the one harnessed by the reported PD-L1 small molecule inhibitors discussed in the current study. They act by stabilizing a non-physiological hPD-L1/h–PD-L1 protein–protein dimer.

### The structural organization Of hPD-L1

Human programmed cell death 1 protein (hPD-L1) is a 290 amino acid long protein that belongs to the B7 family. Structurally, hPD-L1 spans the cellular membrane from inside (a cytoplasmic domain), to the extracellular matrix (an extracellular domain) through a membrane spanning ⍺-helix [30, 72]. Its extracellular domain is composed of two main domains; an Ig-like V-type domain (IgV domain, amino acids 19–127) and an Ig-like C2-type domain (IgC domain, amino acids 133–225). These two subdomains adopt the known sandwich-like β-sheet composition (immunoglobulin fold). The IgV domain of hPD-L1 is the functionally relevant structure of hPD-L1 and is responsible for the binding activities of hPD-L1 to endogenous bio-molecules (*e.g.* hPD-1 and B7-1) as well as exogenous molecules, including antibodies as well as small molecules. For more details regarding the structure and dynamics of the IgV domain of hPD-L1, please consult our recently published paper [30].

Under physiological conditions, there is strong experimental evidence that the extracellular domain of soluble and membrane-bound hPD-L1 exists as a back-to-back dimer [73]. Intriguingly, the hPD-1/h-PDL1 crystal structure showed that it is only the monomeric form of hPD-L1 that forms a complex with hPD-1 [36]. It is not yet obvious, however, whether it is a true or a misleading observation arising from the fact that hPDL-1 in the hPD-1/h-PDL1 co-crystal structure (*e.g.* PDB: 4ZQK) only comprised from the IgV domain, i.e. truncated. Regardless of its oligomeric state, a physiologically active hPD-L1 has its IgV exposing a free interface to bind to hPD-1. In the presence of small molecule PD-L1 inhibitors such as those discussed in this work, the two PD-L1 IgV subdomains form a sandwich-like hPD-L1/BMS/hPD-L1 complex. In this complex the compound binds at the hPD-1 binding interface within hPD-L1, blocking hPD-L1 from binding to hPD-1; hence the name non-physiological dimer.

### Molecular dynamics simulations of a small-molecule bound to hPD-L1 dimers

**Fig. 2:**
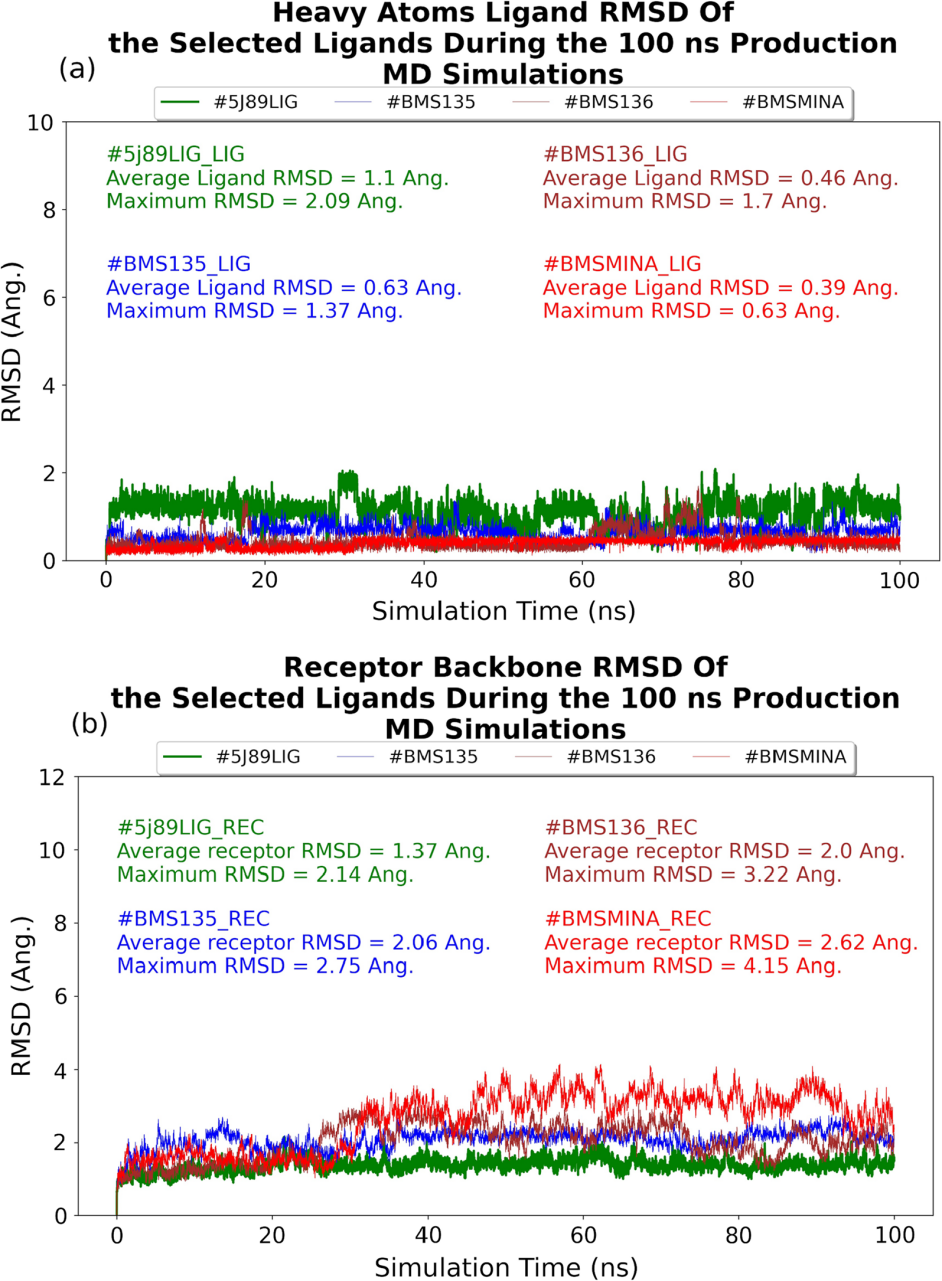
**a** Ligand and **b** receptor RMSD profiles of the studied structural complexes during the 100 ns production MD simulations

Overall, we ran seven MD simulations, comprising five small molecule-bound systems and three protein–protein interaction systems (see “Methods” section for details). To confirm the suitability of the generated MD simulations’ trajectories for further analysis, we first calculated the average root mean square deviation (RMSD) values for all studied systems. To do that we used the first frame of each production simulation as a reference. Our RMSD analysis revealed that the conformational dynamics of the complexes were consistent throughout the entire trajectories (see Fig. 2). For example, the average ligand RMSD values for compounds 5J89LIG, BMS135, BMS136, BMSMINA are 1.1 Å, 0.63 Å, 0.46 Å, and 0.39 Å, respectively. Being a small rigid fragment, BMSMINA showed a stable ligand RMSD value, which was comparable to the rest of investigated compounds. From a protein’s perspective, among all hPD-L1 RMSD values, the BMSMINA system demonstrated the least stability overall with an average protein backbone RMSD value of 2.62 Å and a maximum of 4.15 Å. The average RMSD values for other compounds ranged from 1.37 Å for 5J89LIG to 2.06 Å for BMS135 and their maximum RMSD values ranged from 2.14 Å for 5J89LIG to 3.22 Å for BMS136 (see Fig. 2 for details).

**Fig. 3:**
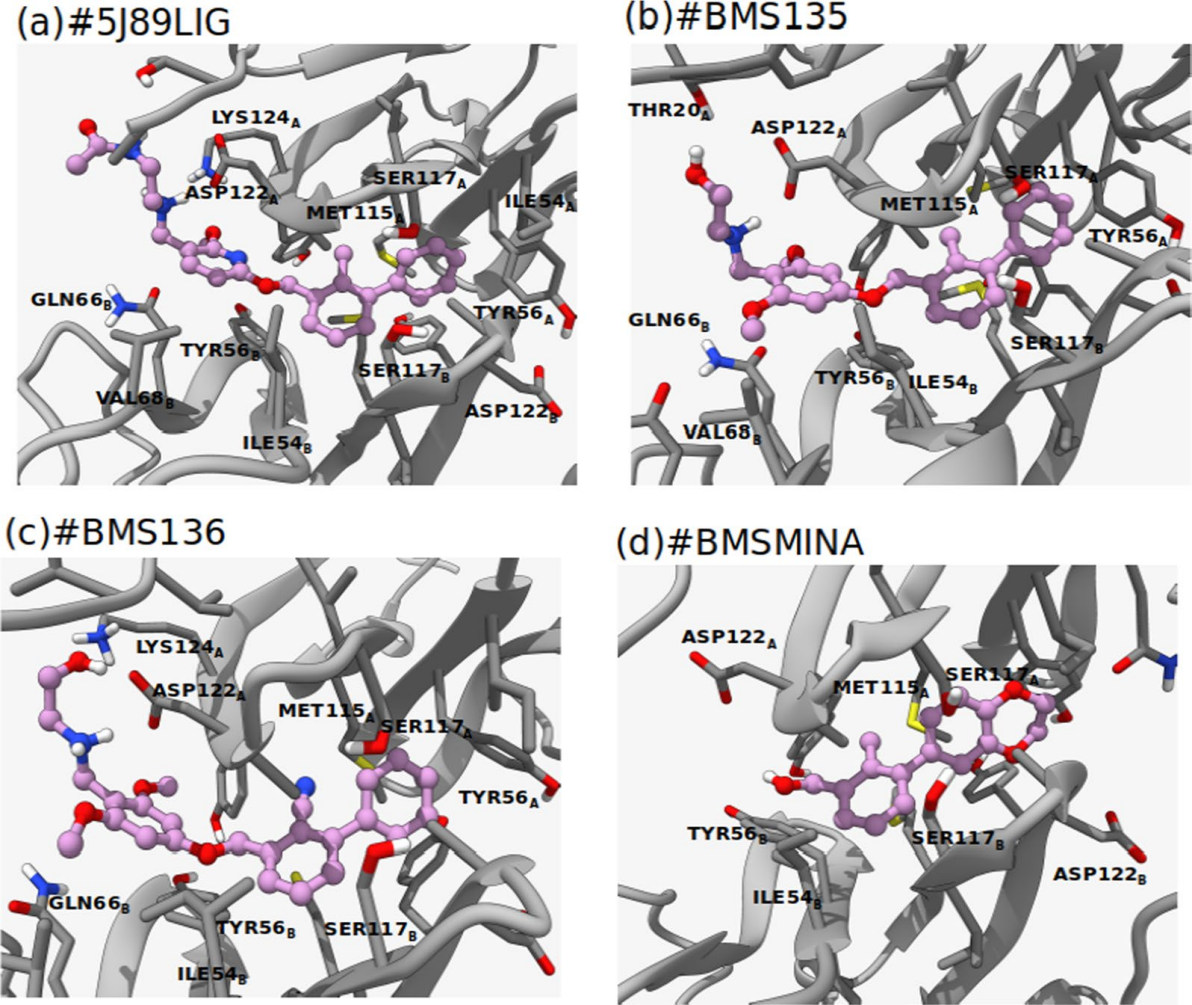
Structural comparison of the protein ligand complexes. **a** The binding geometry of the 5J89LIG ligand in the 5J89 crystal structure, **b**–**d** the predicted binding geometries of the corresponding ligands within the PD-L1 binding sites. Ligand atoms are shown in the blue and sticks representation (carbon: pink, oxygen:red, nitrogen:blue, hydrogen: white), receptor atoms are displayed in the stick representation (carbon: grey, oxygen:red, nitrogen:blue, hydrogen: white)

Figure 3 shows the modes of binding of the studied compounds as predicted by MD simulations. As shown in Fig. 3, for all compounds, the terminal phenyl ring occupies a shallow pocket at chainA formed by residues MET115^A^, SER117^A^, ILE54^A^ and TYR56^A^. The second phenyl ring of the biphenyl ring system is located closer to chainB, interacting with residues MET115^B^, SER117^B^, and ILE54^B^. Although the formed complexes lack the symmetry with respect to the orientation of the hPD-L1 IgV domains, it is evident that the majority of the amino acid residues at the interface surrounding the bound molecules are common between the two hPD-L1 monomers. Additionally, it is obvious that the biphenyl ring system is shared between the two hPD-L1 monomers. This can explain the necessity for the presence of the biphenyl ring system as a minimal structural element required for binding to hPD-L1. The second part of the molecule, (i.e. the methoxy-pyridine-amino-ethyl-acetamide tail) occupies a rather hydrophilic site of the dimer. This hydrophilic tail forms strong H-bonds with the surrounding residues, including ASP122^A^ and LYS124^A^. The pyridine ring is strongly π-stacked with TYR56^B^.

### Free binding energy analysis

**Fig. 4:**
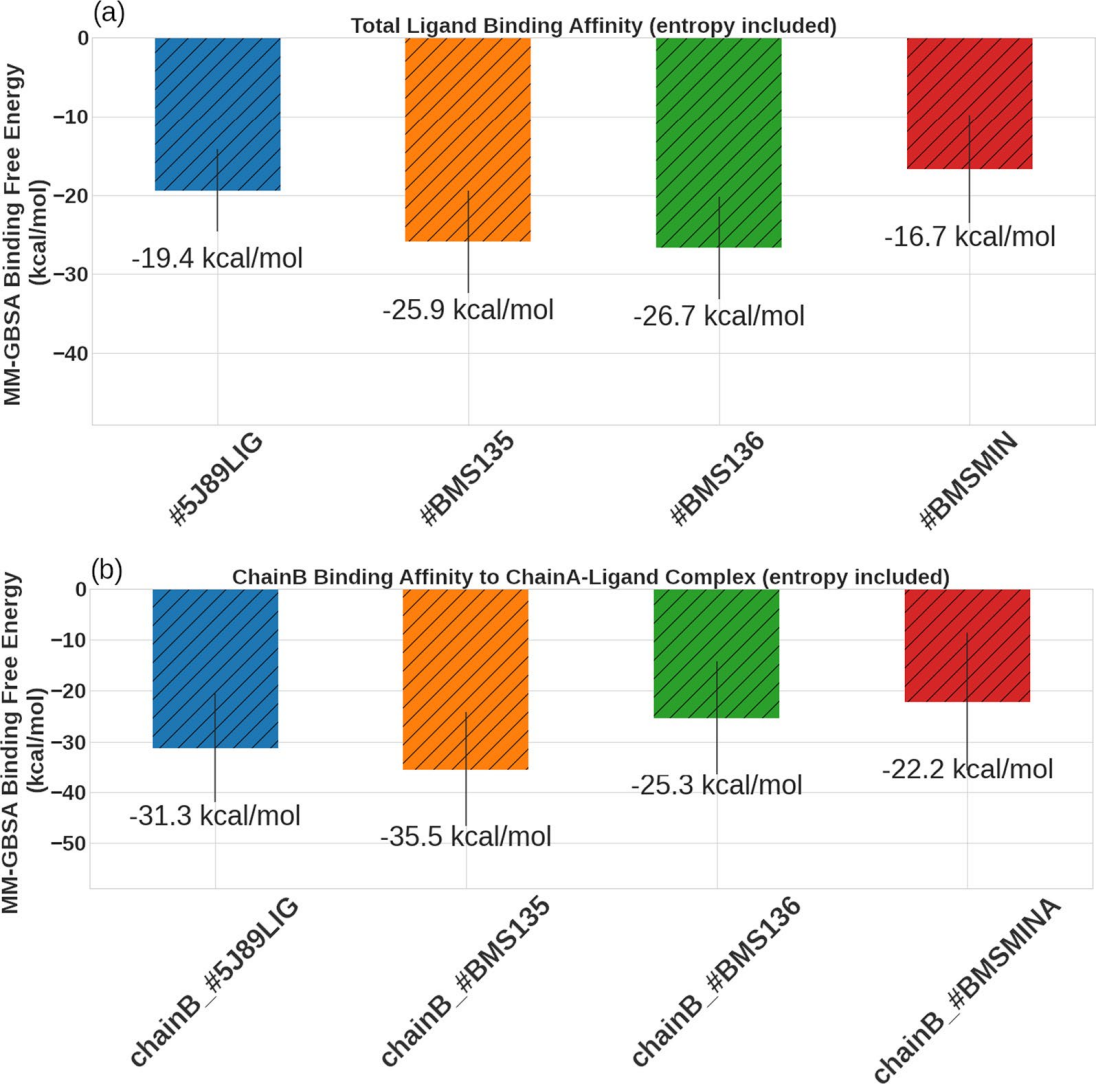
The estimated total AMBER/MM-GBSA (kcal/mol) binding energies of the protein ligand complexes. The binding energies were estimated according to two different scenarios; **a** the small molecule ligand is binding to a receptor composed of the PD-L1 dimer and **b** the second chain of the dimer (chainB) is treated as a ligand whereas chainA complex with the small molecule was treated as the receptor

To shed more light on the binding energetics of the studied molecules, we performed binding energy analyses using the MM-GBSA approach (see “methods” section for details). As shown in Fig. 4a, the total binding free energy values of the different ligands under study are quite comparable. All ligands exhibited binding affinities less than − 15 kcal/mol. While the minimum biphenyl fragment (#BMSMINA) showed a binding energy of − 16.7 ± 6.8 kcal/mol, as expected, all other larger ligands (*i.e.* #5J89LIG, #BMS135, #BMS136) showed better binding affinities of less than − 19.0 kcal/mol, with compound #BMS136 shows the lowest binding affinity that is given by − 26.7 ± 6.5 kcal/mol.

The above free energy analyses assumed that the small molecules are bound to a hPD-L1 dimer, stabilizing the interactions of the two monomers. However, it is also possible that a small molecule can bind to this dimer in a different scenario. That is, a small molecule can initially associate with a PD-L1 monomer and this transient complex and then recruit another PD-L1 monomer to seal the binding site and form a stable small molecule-sandwiched PD-L1 dimer. To mimic this scenario, and to study its free energy of binding, we re-calculated the MM-GBSA scores of the different complexes, after considering one a monomer (*e.g.* chain A of hPD-L1) bound to a small molecule as a receptor and the second hPD-L1 chain (*e.g.* chain B) as the ligand. This is illustrated in Fig. 4b, which shows the association of chain B with chain A of hPD-L1 in the presence of any of the small molecules (*i.e.* #5J89LIG, #BMS135, #BMS136, and #BMSMINA). For example, the presence of #BMSMINA lead to a free energy of − 22.2 ± 13.5 kcal/mol for the hPD-L1 dimer formation, indicating that the minimal active fragment that occupies the symmetric pockets on the surfaces of the two hPD-L1 chains is sufficient to induce a stable dimer formation. Any further improvement to the binding free energies of the formed complexes is due to the extra stabilization gained from the interactions of the additional pharmacophoric features in the larger small molecules with that present in hPD-L1 surface (as described above). For example, in the presence of compound #5J89LIG, the free energy was estimated by − 31.3 ± 10.8 kcal/mol (*i.e.*, an improvement of ~ 11 kcal/mol from that of #BMSMINA). Similarly, the estimated binding free energies for the complexes involving #BMS135, and #BMS136 are − 35.5 ± 11.3 kcal/mol, and − 25.3 ± 11.07 kcal/mol, respectively. From the perspective of chainB recruitment to chainA-ligand mechanism of complex formation, the free binding energy is consistent with our recent IC_50_ measurements that showed #BMS135 to be a better inhibitor for the h-PD1/h-PD1 interaction with an IC_50_ that is given by 79.1 nM compared to #BMS136 with an IC_50_ of 96.7 nM. To the best of our knowledge, no IC_50_ measurement was conducted for #BMSMINA, which is expected to be a weak inhibitor anyway being a small fragment. Whether the agreement is a support of the proposed mechanism of complex formation or just a mere coincidence requires simulating a large number of ligands with known inhibition data and this will be the focus of a future work.

### Interactions of PD-L1 chains in the absence of a small molecule

It is well-known that the affinity and specificity of PPIs are often determined and enhanced by the levels of geometric and chemical complementarities of two proteins. In particular, the electrostatic forces are considered as important factors in protein–protein complex formation [74–77]. In the case of immune checkpoints proteins such as hPD-L1, their surfaces display multiple characteristics such that they could interact with the same type of proteins on one side to form oligomer complexes. They can interact with other protein partners (*e.g.* hPD-1 in the case of hPD-L1) on the opposite face to form heteromeric complexes. However, the small molecules studied here tend to induce a dimerization of two hPD-L1 monomers on their heteromeric complexation faces, thereby preventing a normal hPD-1/hPD-L1 interaction. While our MD simulations-based binding free energy analyses presented above was able to identify key residues on the hPD-L1 surface that are involved in small molecule-induced dimerization, it is not clear which mechanism is preferred for such binding. That is whether the small molecule inhibitor binds to an existing hPD-L1: hPD-L1 dimer, or it binds first to a hPD-L1 monomer and then attracts another monomer to form a dimer complex. To answer this question, we studied the interactions of an apo hPD-L1 dimer (i.e., a hPD-L1 dimer structure obtained after removing the small molecule from the complex) using 75 ns long MD simulations. During these simulations, we applied a physical restraint of 0.5 kcal mol^−1^ Å^−2^ on the backbone of the residues forming the GF strands (*i.e.* residues 33–42 and 93–105) in the two PD-L1 chains. Since these minimal restraints were applied on the face opposite to where the small molecule binds (see Additional file 1: Fig. S1a), these restraints help compensate for the loss of the bound small molecule, which mediates the binding of and tether the surfaces of two hPD-L1 chains at a close distance during the simulation.

**Fig. 5:**
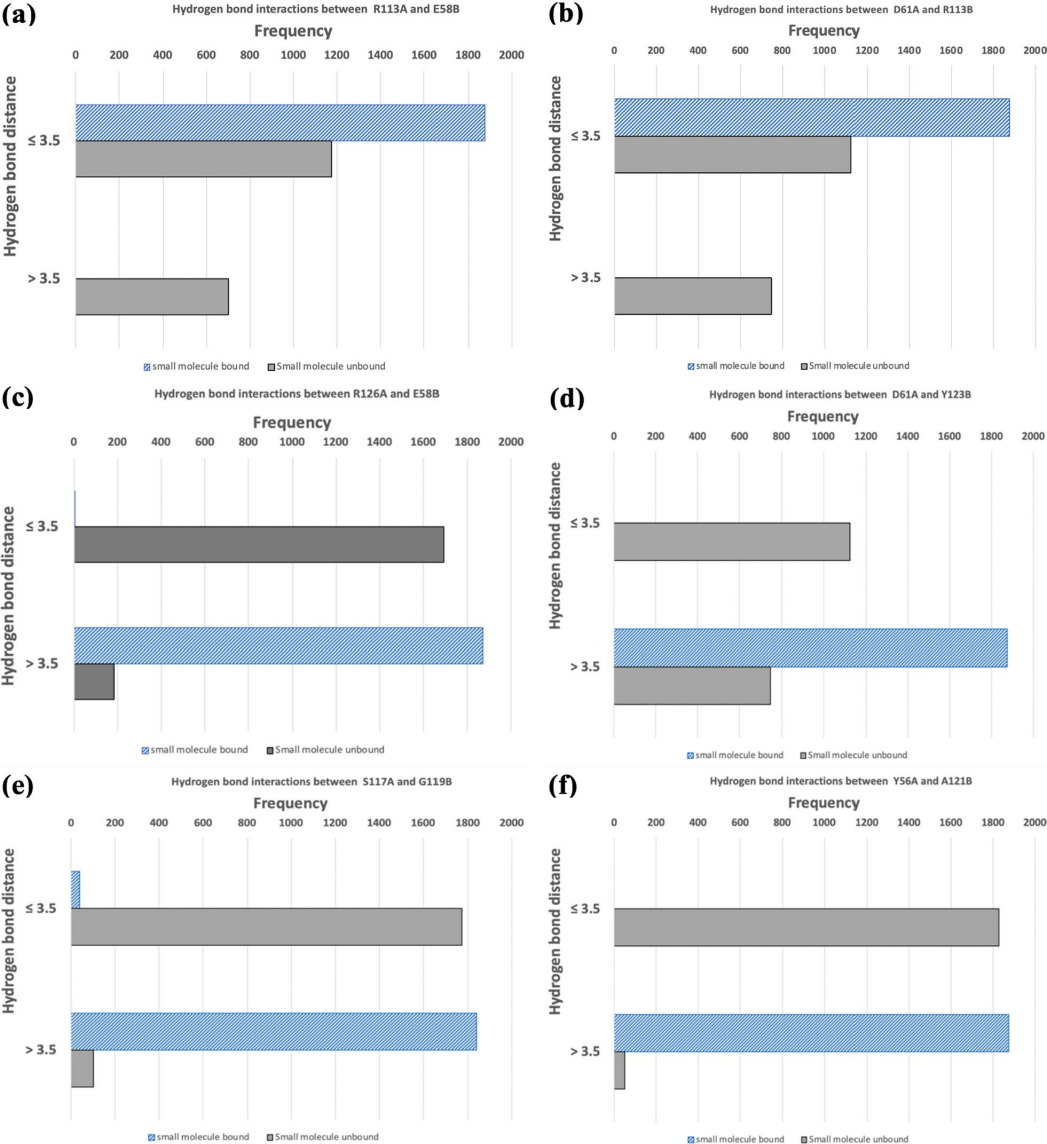
The analyses of key hydrogen bond (H-bond) interactions in the small molecule-bound and unbound systems. The frequency of H-bond distances staying within or over a 3.5 Å threshold in different residue-residue pairs such as ARG113^A^/GLU58^B^ (**a**), ASP61^A^/ARG113^B^ (**b**), ARG126^A^/GLU58^B^ (**c**), ASP61^A^/TYR123^B^ (**d**), SER117^A^/GLY119^B^ (**e**), and TYR56^A^/ALA121^B^ (**f**) are provided. Single letter codes of amino acids are used in the figure

Initially, we tested the stability of the apo-systems using MD simulations. The backbone RMSD of the restrained apo-hPD-L1 dimer was almost similar to that of a ligand-bound hPD-L1 complex with an average RMS fluctuation of ~ 0.26 nm (see Additional file 1: Fig. S1b). We then analyzed the interactions of hPD-L1 chains (through their heteromeric complexation faces) in the absence of a small molecule and compared them against those in a small molecule-bound complex (see Fig. 5 and the Additional file 1: Fig. S2). Our analyses indicated that two strong salt bridge interactions mediated by ARG113^A^/GLU58^B^ (see Fig. 5a) and ASP61^A^/ARG113^B^ (see Fig. 5b) made significant contributions to the stability of the bound systems (see in Fig. 2). These salt bridges are weakened by the absence of a small molecule in the apo-system. As shown in Fig. 5a, in the small molecule-bound system, the hydrogen bond (H-bond) distance between ARG113^A^ and GLU58^B^ throughout the simulation remains at distance less than 3.5 Å (a generally accepted threshold for H-bonds) [78]. However, the frequency of the ARG113^A^/GLU58^B^ H-bond within this threshold (*i.e.*, ≤ 3.5 Å) was reduced by at least 40% in the apo simulation. Similar behaviour was observed in the H-bond distance for the ASP61^A^/ARG113^B^ pair (see Fig. 5b), where the frequency of H-bond interactions between these two residues fell by ~ 40% in the simulation of apo complex. On the other hand, in the absence of a small-molecule intervention, the two hPD-L1 chains established unique inter-protein H-bonds that were not seen in the small molecule-bound complex. For example, H-bonds within the two residual pairs such as ARG126^A^/GLU58^B^ (see Fig. 5c) and ASP61^A^/TYR123^B^ (see Fig. 5d) were only seen in the absence of a bound small molecule. This is evident from the clear shifts in the H-bond distances of the two pairs in the small molecule-bound and unbound complex; the distances mostly stayed > 3.5 Å threshold in the former whereas it predominantly dropped within the threshold in the case of the latter (see Fig. 5c, d). In addition, two new H-bonds such as SER117^A^/GLY119^B^ (see Fig. 5e) and TYR56^A^/ALA121^B^ (see Figs. 5f) were found to be strongly formed between the hPD-L1 chains in the absence of a small molecule. It is important to note that some of the residues that are involved in forming these H-bonds are only interacting in the apo complex. This includes TYR56, TYR123, and ALA121, which exhibited significant contributions in the interactions with the bound small molecules (see Fig. 3). Taken together, our restrained MD simulations suggest that it is not possible for a small molecule to bind to an existing PD-L1 dimer (see Additional file 1: Fig. S2) and, therefore, it is hypothesized that a small molecule most likely binds first to a hPD-L1 monomer and subsequently attracts another soluble monomer to form a stable dimer complex.

### Preferential binding of hPD-L1 to other protein partners

**Fig. 6:**
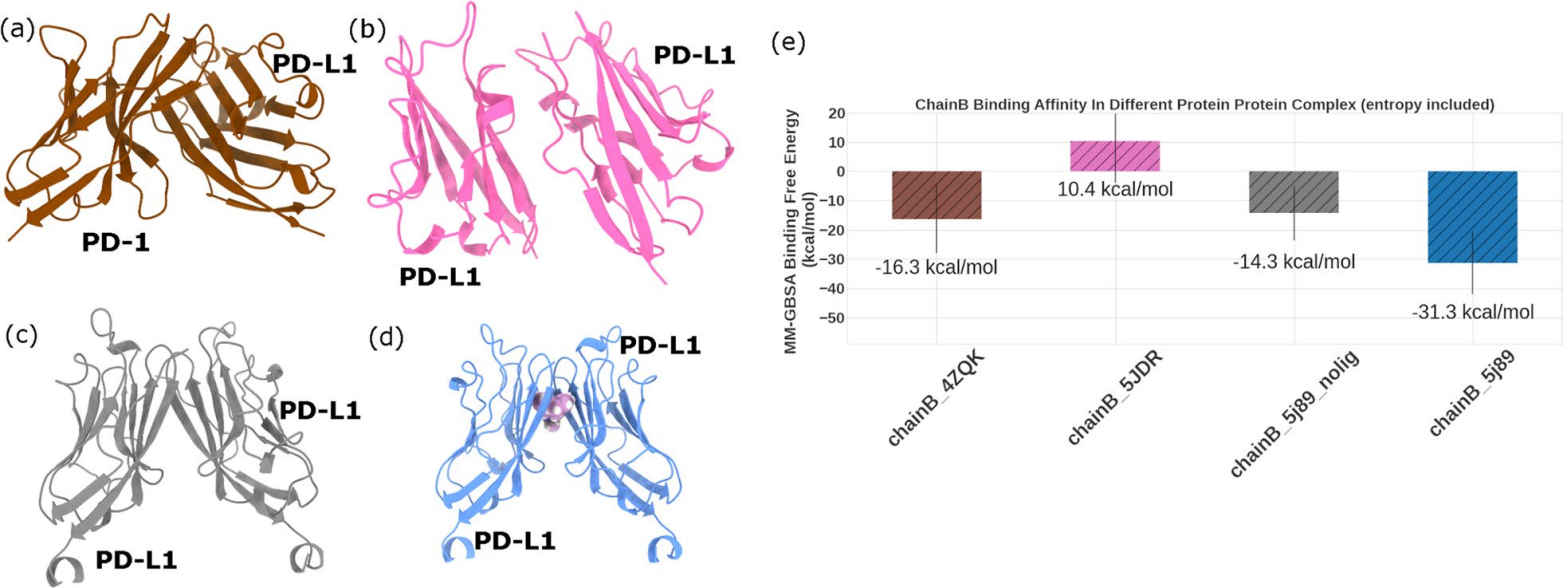
Calculated binding energies of the PD-L1 protein in different protein–protein or protein–ligand complexes. The most stable complex of PD-L1 was found to be its complex with the PD-L1 small molecule complex (**d**), followed by its complex with PD-1 (**a**). The panel (**e**) represents the estimated binding affinities for each complex using MM-GBSA

At the molecular level, the biological environment is a densely packed crowd of thousands of bio-macromolecules, with a high opportunity of frequent encounters [79]. Many of these encounters are non-specific in nature and do not lead to any physiological function. In particular, multiple potential protein partners, including hPD-L1 itself, hPD-1, B7-1 and more, may surround a hPD-L1 monomer. The balance between free hPD-L1 and its affinity towards such potential binding partners firmly regulates the physiological functions of hPD-L1. To investigate the preference of hPD-L1 to bind any of these potential partners, we estimated the affinities of a hPD-L1 monomer to both hPD-L1 and hPD-1 in different settings. This included testing a hPD-L1/hPD-1 complex, a physiological back-to-back hPD-L1/hPD-L1 complex, and the non-physiological, small molecule-mediated face-to-face hPD-L1/hPD-L1 dimer with and without bound small molecules. As discussed in the methods sections, the starting conformations of all these complexes were adopted from their corresponding crystal structures. The simulations were then performed for 120 ns and the last 100 ns of the simulations’ trajectories were used for analysis. Cartoon representations of these complexes are shown in Fig. 6a–d.

Figure 6e shows the estimated MMGBSA binding free energies for hPD-L1 in complex with different protein partners. The data represents the binding affinities of hPD-L1 in two physiological complexes, (a) with hPD-1 and (b) with hPD-L1 (*i.e.* the back-to-back dimer). The figure also shows the estimated energies for two non-physiological complexes induced by a small molecule in the absence of the bound molecule (c) and in the presence of the bound molecule (d). As shown in Fig. 6, it is clear that hPD-L1 has a strong preference to form a protein–protein complex in the presence of a bound small molecule over other potential complexes. The binding energies of hPD-L1 in the studied complexes adopt the following trend: hPD-L1/BMS/hPD-L1 <  < hPD-L1/hPD-1 < hPD-L1/hPD-L1 (non-physiological protein dimer) <  < hPDL1/hPDL1 (physiological protein dimer). The average MMGBSA binding free energies of the hPD-L1/BMS/hPD-L1 complex is estimated to be − 31.3 ± 10.8 kcal/mol. That is almost 15 kcal/mol stronger than that of the hPD-L1 in complex with hPD-1, which is estimated to be − 16.3 ± 11.7 kcal/mol. This is followed by the hPD-L1/hPD-L1 in the BMS bound-like complex (without the small molecule, *i.e.* non-physiological protein dimer), that was found to be − 14.2 ± 9.3 kcal/mol, and finally the physiological protein dimer (back-to-back) that was found to be unfavorable, 10.4 ± 14.4 kcal/mol. This data is consistent with the expected ranking of binding strengths of hPD-L1 in the different protein–protein complexes. The Kd value of hPD-1/hPD-L1 binding is reported as 8.20 ± 0.10 μM [72]. As recently disclosed, the IC_50_ value of the small molecule induced hPD-L1/hPD-L1 dimer is generally in the nano-molar range for efficient inhibitors. Note that entropy estimation was included in this calculation through normal mode analysis. The lowest binding affinities achieved in the hPD-L1/BMS/hPD-L1 demonstrates that, at least in theory, BMS small molecules are capable of breaking down the preformed immune-inhibitory hPD-L1/hPD-1 complex, which can eventually lead to a reactivation of the immune system to fight against cancer. Other contributing factors to the structural preference may be the expression level of the different proteins in different biological contexts.

### Computational water mapping through GIST and HSA

For many computational structural biologists, modeling a biomolecular system in explicit water has a strong appeal over implicit solvent modelling. This mainly due to the ability to observe and monitor water-mediated interactions throughout an MD simulation in an explicit water setting. With the advent of robust computational infrastructure (computing clusters and algorithms) capable of performing explicit water simulations at a relatively short time scale, explicit methods are gradually replacing the implicit methods for solvent mapping. A clear example showing the “fall of the implicit water empire” has been recently demonstrated in a study by Bucher et al. [80]. In this study, four different commercially available solvent mapping tools (SZMAP, WaterFLAP, 3D-RISM, and WaterMap) were compared for their ability to correctly identify the critical hydration sites in three different protein targets relevant to drug discovery problems in industrial settings. In all the studied examples, the simulation(explicit)-based approach, exemplified by WaterMap [81], from Schrodinger, offered a clear advantage over existing grid(implicit)-based approaches. In particular, the authors highlighted the fact that certain water-solute, or water-mediated H-bonding interactions, which are obviously lacking in the grid-based approaches, are indispensable for correctly identifying the critical hydration sites.

Solvent mapping enables researchers to classify water molecules surrounding the protein into two categories. The first category includes energetically unfavourable water molecules (*i.e.* unhappy water) and second includes those water molecules that are hard to displace (*i.e.* energetically favourable, happy water) [82]. One can use the information gained from the solvent mapping analysis to rationally perform further rounds of lead optimization, virtual screening, selectivity analysis, ligand pose prediction, and druggability assessments. In many cases, bound water molecules are extremely hard to displace by a small molecule [83, 84]. Furthermore, relocating enthalpically favorable solvating water molecules from the binding site to bulk solvent has been reported as the rate-limiting step for ligand–protein binding in many protein targets [85]. One can exploit such water molecules to engineer an additional interaction with the target or decide to ignore them and look for other innovative solutions.

Given the exceptional potency of the reported hPD-L1 inhibitors (*e.g.* BMS compounds) in facilitating the binding of two hPD-L1 monomers, we examined the role of water in triggering this interaction. Towards this goal, we performed a 100 ns restrained explicit water MD simulation for the free hPD-L1 protein in a box of TIP3P water. The generated MD trajectory was further analyzed using GIST and HSA to identify critical hydration sites at the surface of the hPD-L1 protein. The output of this analysis, a csv file, was further processed using ambertools and a score (***K***_***ss***_) was generated to quantify the extent of hydration site favorness, taking the mean water-water interaction energies of bulk water, and the site occupancies with water molecules into account. The full output of HSA combined with the ***K***_***ss***_ scores is listed in Table 1.

**Table 1:** Thermodynamic properties for water hydration sites

Site index	Occupancy	Esw	Eww	Etot	TSsw_trans	TSsw_orient	TStot	Kss_Score
W13	0.56	− 2.73	− 5.87	− 8.60	1.09	− 0.98	0.11	0.52
W3	0.99	− 4.63	− 4.38	− 9.01	2.20	− 1.86	0.33	0.51
W28	0.34	− 1.72	− 7.00	− 8.72	0.66	− 0.43	0.23	0.27
W14	0.52	− 2.41	− 6.87	− 9.28	1.02	− 0.77	0.25	0.13
W26	0.35	− 1.28	− 7.92	− 9.20	0.62	− 0.54	0.08	0.11
W16	0.49	− 3.98	− 5.33	− 9.31	1.23	− 0.93	0.31	0.11
W23	0.45	− 3.33	− 6.33	− 9.66	0.92	− 0.91	0.00	− 0.06
W29	0.32	− 0.74	− 9.08	− 9.83	0.60	− 0.40	0.19	− 0.10
W31	0.29	− 8.19	− 1.68	− 9.87	1.01	− 1.03	− 0.01	− 0.10
W30	0.28	− 1.57	− 8.34	− 9.91	0.54	− 0.63	− 0.08	− 0.11
W12	0.59	− 7.43	− 2.33	− 9.76	1.95	− 2.25	− 0.30	− 0.13
W25	0.34	− 4.94	− 5.13	− 10.07	0.81	− 1.00	− 0.19	− 0.18
W10	0.69	− 6.03	− 3.79	− 9.82	1.54	− 1.29	0.25	− 0.20
W17	0.48	− 3.17	− 6.84	− 10.01	0.90	− 1.16	− 0.26	− 0.23
W21	0.43	− 12.59	2.45	− 10.14	1.46	− 1.50	− 0.04	− 0.26
W18	0.48	− 3.63	− 6.47	− 10.10	1.08	− 0.50	0.59	− 0.27
W24	0.4	− 2.96	− 7.46	− 10.42	0.98	− 0.46	0.53	− 0.36
W19	0.45	− 7.74	− 2.79	− 10.54	1.09	− 1.27	− 0.17	− 0.45
W27	0.37	− 9.48	− 1.46	− 10.94	0.96	− 1.49	− 0.52	− 0.52
W7	0.81	-8.44	− 1.92	− 10.36	1.69	− 1.55	0.14	− 0.67
W15	0.5	− 8.53	− 2.45	− 10.98	1.27	− 1.27	0.01	− 0.72
W20	0.45	− 9.11	− 2.07	− 11.18	1.23	− 1.64	− 0.41	− 0.74
W11	0.67	− 8.69	− 2.23	− 10.91	1.56	− 2.04	− 0.48	− 0.93
W8	0.8	− 4.73	− 6.01	− 10.74	1.60	− 2.27	− 0.68	− 0.97
W9	0.77	− 6.54	− 4.30	− 10.84	2.13	− 2.56	− 0.43	− 1.01
W22	0.43	− 13.37	0.82	− 12.55	1.29	− 2.04	− 0.75	− 1.30
W5	0.95	− 6.73	− 5.82	− 12.55	1.94	− 2.72	− 0.79	− 2.87
W2	1	− 15.69	1.85	− 13.83	2.44	− 2.99	− 0.55	− 4.30

**Fig. 7:**
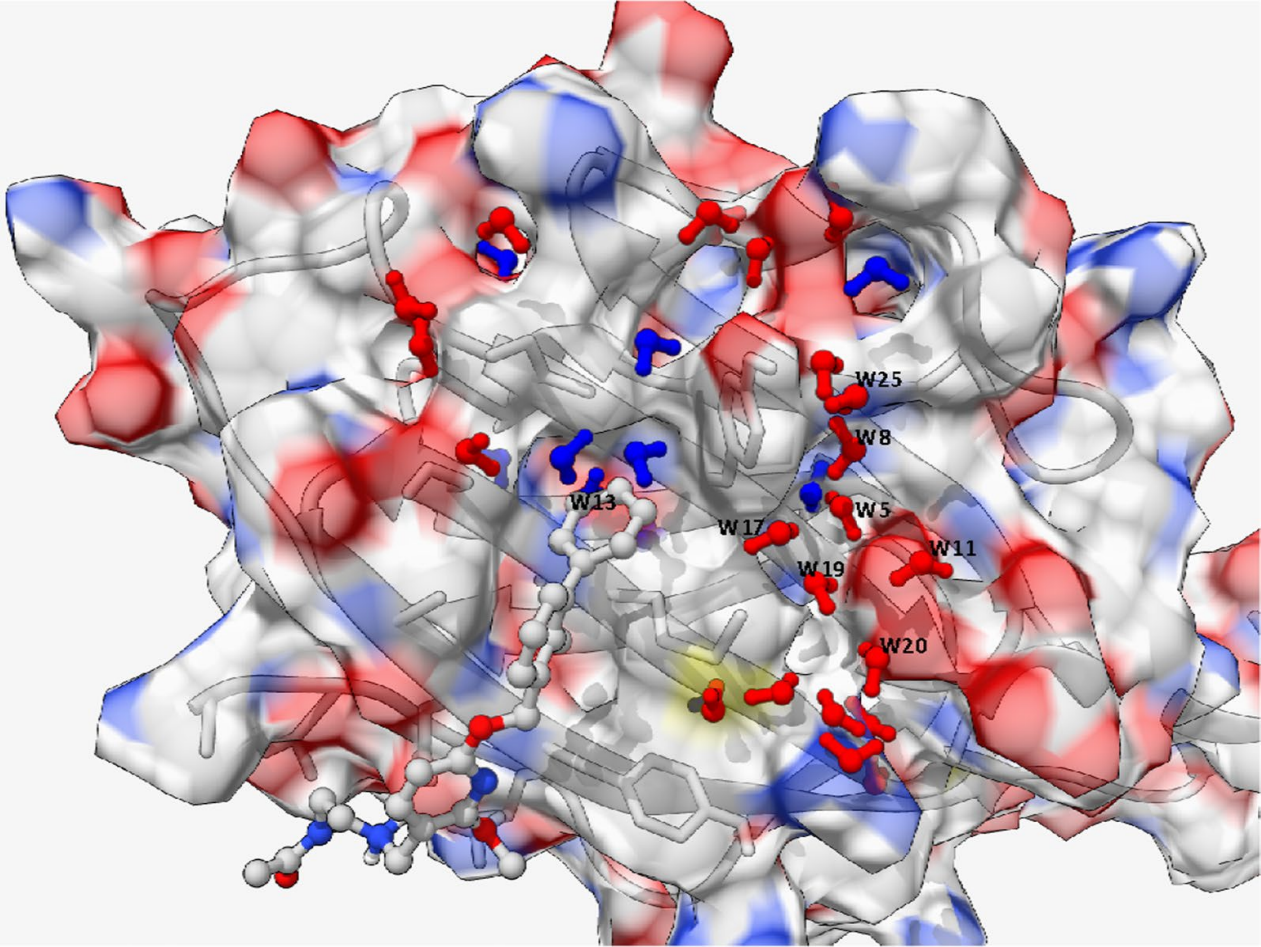
Computational solvent mapping analysis for the PD-L1 protein using GIST over 100 ns restrained MD simulation. Water molecules are coloured according to the sign of their corresponding ***K***_***ss***_ scores where blue colour means energetically unfavourable hydration sites (unhappy/easy to displace water) and red coloured waters designates the favourable hydration sites (happy/hard to displace water). The 5J89LIG small molecule is overplayed to the structure to provide a direct structural interpretation of the hydration site. Surface residue patches are coloured according to their corresponding properties, where red patches indicate negatively charged residues, blue patches indicate positively charged residues, grey patches indicate neutral residues

As shown in Table 1, our analysis identified 32 potential hydration sites surrounding the surface of hPD-L1. Table 1 provides a complete list of all these sites and their associated thermodynamic quantities. For a visual representation for some important sites at the surface of the hPD-L1 protein, Fig. 7 displays the identified hydration sites using the most probable water configuration at each site as a static water molecule. The designated water molecules are colored according to their corresponding ***K***_***ss***_ scores, where water molecules with positive scores (***K***_***ss***_ > 0, unfavorable hydration sites) are colored in blue, and water molecules with negative *K*_*ss*_ scores (favorable hydration sites) are colored in red. In the same figure, the co-crystallized pose of #5J89LIG is overlaid on the surface of the protein to provide a direct structural insight, and the protein surface is colored by heteroatoms, whereas grey surface means carbon atoms (usually associated with lipophilic residue batches).

A closer look at Fig. 7 shows that a ligand does not interfere with any of the tightly bound water molecules (*i.e.* energetically favourable, red coloured). Furthermore, one of the most energetically unfavourable water molecules, W13 (*K*_*ss*_ = 0.52) is positioned exactly at the tip of the binding cleft that accommodates the phenyl ring of the small molecule ligand. Other high-energy water molecules are occupying nearby surface cavities. On the contrary from these enthalpically unfavourable waters, a trail of energetically favourable waters (W5, W8, W9, W11, W17, W19, W20, W25) is occupying the C–C′ turn at the surface of hPD-L1. As also shown in Table1, these waters take advantage of a strong interaction with a surrounding group of charged residues, such as GLU58, ASP61, ARG113 and ARG125. These water molecules also form local water clusters, hence, lowering their total energies while making these clusters very energetically favourable. In a recent x-ray crystallography study by Zhang et al. some of these sites have been shown experimentally to stabilize the hPDL1 complex with an engineered nanobody (KN035). For example, the Zhang study unambiguously identified the involvement of a bridging water molecule to stabilize the polar interaction between ASP61 on the surface of hPD-L1 with SER108 on the surface of the KN035 nanobody [38]. A similar observation was also made for the interaction of hPD-L1 with hPD-1, where a strong water-mediated interaction was observed between the carboxyl group of GLU58 on the surface of hPD-L1 and the carbonyl group of carbonyl of ILE134 and on the surface of hPD-1 [36].

In a typical scenario and ignoring the impact of the presence of a second protein chain to sandwich the small molecule ligand, extending the small molecules to the energetically favourable hydration sites will result in a significant reduction in the overall affinity of the small molecules to the protein target. On the other hand, extending the terminal phenyl ring by additional substitution, as is the case in new BMS derivatives, should enhance the binding affinities, as it will lead to displacing unfavourable water molecules to the bulk. Some of the more recent PD-L1 inhibitors empirically exploited this fact through extending the biphenyl ring by an additional alicyclic 1,4-dioxane ring, as in the #BMSMINA fragment.

### 2D QSAR analysis

**Fig. 8:**
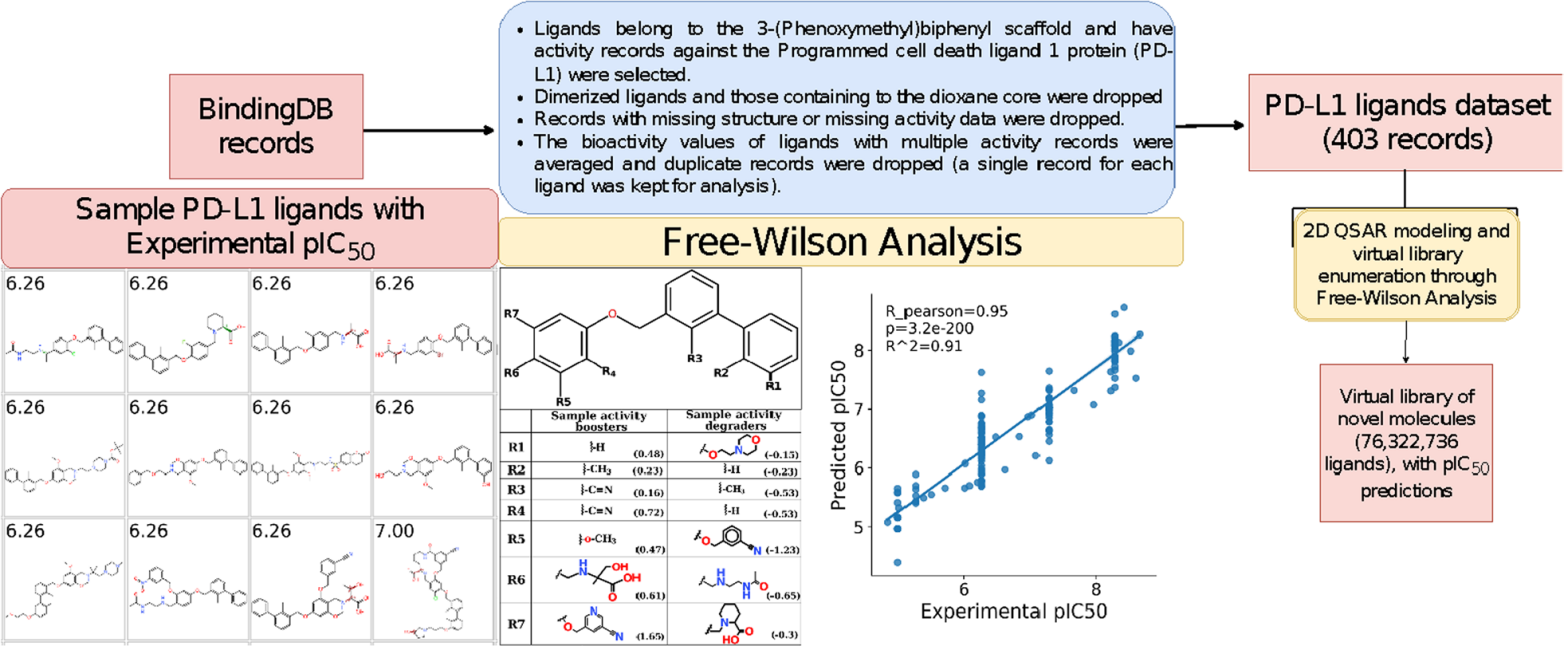
A diagram summarizing the steps of the entire ligand-based analysis workflows, including the Free-Wilson analysis

To augment the structural data with a more generalized data-driven analysis, we employed a 2D QSAR approach to study the entire BMS series of ligands listed in the literature. A diagram representing the developed 2D QSAR workflow is shown in Fig. 8. We primarily focused on the 3-(Phenoxymethyl)biphenyl series as a prototype. This series also has the largest number of annotated activity records in the literature. Bioactivity data of 403 unique ligands were collected from the BindingDB database. The full dataset is given in the Additional file 2: as a CSV file (S3). The IC_50_ values were transformed to the log scale (*i.e.* pIC_50_). Figure 8 shows the chemical structures of a few sample ligands with their corresponding measured experimental pIC_50_ values. The model was built through the ridge regression protocol and achieved Pearson correlation (R) value of 0.95, and a 0.91 for the coefficient of determination (R^2^) value. A scatter plot of the regression model showing the experimental versus the predicted pIC_50_ values is given in Fig. 8.

The functional groups with maximum coefficients on the positive and negative sides of the Free-Wilson analysis were selected for further investigation (full results are given as Additional files CSV files; Additional file 3: S4: R-groups decomposition, and Additional file 4: S5: R-groups coefficients). Positive coefficients denote activity boosters, whereas negative values denote activity degraders. As shown in Fig. 8, it is clear that a maximum activity gain or loss originates from substituents at the terminal phenoxy group, namely, the R4, R5, R6, and R7 sites. For the R5 and R7 sites, an optimum activity gain is achieved by the methoxy and the methoxy-cyanopyridine groups with a coefficient of 0.47 and 1.65, respectively. A substitution with the bulky methoxy-cyanophenyl at the R5 site results in a significant loss in the biological activities (coefficient = − 1.23). The R7 site seems to be sensitive to charged substitution where the piperidine-carboxylate group results in a significant reduction in the biological activities (coefficient = − 0.3). The R6 site, on the contrary, has a strong preference for a charged hydrophilic functional group, for example, the alkylamino-3-hydroxy-2-methyl propionate functional group (coefficient = 0.61) probably as a result of the engagement of this group with hydrophilic residues cluster at the PD-L1/PD-L1 interface. Please note that the nature of interaction at this site is both direct electrostatic as well as water-mediated interactions as the site itself is solvent-exposed; this gives rise to potential charge-assisted interactions of any signs, positive or negative. Non-charged terminal motif substitution at R6 seems to deteriorate the activity of the series where the N-alkyl acetamido group exhibited a coefficient of -0.65.

Substitutions at the biphenyl core (*e.g.* R1, R2, and R3) exhibited a distinct pattern. The R1 site seems to have more preference for the unsubstituted derivatization, with substitution with hydrogen gives a coefficient of 0.48. For substitutions at the R2 and R3 sites, it seems that only small substitutions have been explored at these two sites. This is not surprising given the relatively small allowed volume of the binding cavity, where the biphenyl core is perfectly sandwiched between the two PD-L1 IgV monomers. There is a clear advantage of the methyl substitution at the R2 site (coefficient = 0.23 versus − 0.23 for the unsubstituted site). For the R3 site, the nitrile group gives a moderate preference (coefficient = 0.16) versus the methyl group substitution (coefficient = − 0.53). The SP2 nitrile group is a known powerful water displacing motif [86, 87] and based on our hydration analysis (see above), displacing binding site water at this site is a prerequisite for binding in this class of PD-L1 dimerization inducers. This could explain the preference of the cyano group at the R3 site compared to the methyl group. It was also interesting to find no unsubstituted biphenyl derivatives existed within the analyzed set of inhibitors, particularly at the R3 site. Although this could be a limitation in the selected datasets, a more convincing explanation seems to be that the non-coplanarity of the biphenyl core is the conformationally preferred structure to induce PD-L1 dimerization and is essential for activity. Clearly, the R2/R3-ortho-ortho substitution is a trigger for this non-coplanarity that results from steric hindrance. Other common substitution patterns on the biphenyl core, such as the para–para and para–ortho will eventually lead to linear or disc-like molecules [88]. As of August 2021, there have been approximately 11 protein–ligand co-crystal structures from the 3-(Phenoxymethyl)biphenyl anti-PDL1 series deposited in the PDB. Nevertheless, we could not find a single protein–ligand crystal structure from this series where the biphenyl core is unsubstituted at the R3 site. The biphenyl fragment is a very common fragment in drug/drug-like molecules, and is usually introduced into organic compounds through the renowned Suzuki–Miyaura coupling reaction. Notably, this fragment was also the core fragment that inaugurated the discovery of a very potent class of direct-acting NS5A inhibitors that are clinically used for hepatitis C Virus (HCV) treatment [89]. Daclatasvir, the prototype of this class of direct-acting NS5A inhibitors and is one of the best-known biphenyl-containing drugs, and its analogs are believed to target the dimeric form of the NS5A protein [46, 90]. Whether there is something special about the biphenyl core making it a preferred binding partner for dimer-forming proteins, or this is a mere coincidence is a question that is worth further investigation. As such, one can suggest including ligands containing the biphenyl core fragment as an essential component of focused chemical libraries that aim at targeting dimer-forming proteins. This is a question to be answered in future research by our group and others.

Given the strong coefficient of determination (R_squared = 0.91) obtained from the regression model, we moved ahead and enumerated a virtual library of unexplored derivatives (76,322,736 molecules) with all possible R-groups permutations and predicted their pIC50 values. A random subset of ligands that achieved a predicted pIC_50_ values >  = 8 is given in Additional file 5: (S6).

## Conclusions

This paper aims at answering a few fundamental questions regarding the specific molecular interactions responsible for triggering the formation of the non-physiological, small molecules induced PD-L1 dimer. By conducting a blend of structural and ligand-based analyses, we were able to investigate the role played by surface residues, the role of desolvation and functional group substitutions in the observed potencies of anti-PD-L1 ligands bearing the phenoxy-methyl biphenyl core. Our molecular dynamics simulations and binding free energy analyses revealed several interesting observations. The data explains the reasons for the need of a biphenyl core to be shared among the two PD-L1 monomers to trigger complex formation. Furthermore, the binding energy decomposition analysis highlighted the possible role of the cluster of charged, salt-bridge forming residues at the G,F,C,C′ strands of the surface of the PD-L1 protein as the main driving forces for the formation of the protein–ligand-protein complex. The performed computational solvent mapping analysis revealed that the first step of the molecular association involves the desolvation of a highly energetic hydration site at the centre of the hPD-L1 interface.

Our ligands-based analyses revealed the importance of each reported substitution around the biphenyl-core scaffold. For example, substitutions around the terminal phenoxy group are more diverse than those allowed around the biphenyl core. The cyano group substitution at the R3 site seems to be more favourable than methyl group substitution. Substitution at the R3 site seems to be mandatory for activity, presumably as a trigger to ensure the non-coplanarity of the biphenyl core. The Free-Wilson enumerated chemical library could provide an additional mine of potentially active molecules and the library (~ 76 M) molecules is available free of charge for academic purposes.

We finally hope that the data presented here can foster the ongoing research efforts aiming at finding small molecule drugs active against immune checkpoint receptors and the immuno-therapy drug discovery field in general.

## Supplementary Information

**Additional file 1: Figure S1.** The 3D structure of a small molecule free PD-L1 dimer (a) and the comparison of backbone RMSD fluctuations in small molecule-bound and unbound systems during 75 ns long MD simulation (b). (a) In the 3D structure of the PD-L1 dimer shown as a cartoon representation, the chain A is shown in blue and chain B in Red. The region corresponding to the residues 33-42 and 93-105 in both the chains, where a physical restraint of 0.5 kcal/mol were applied, are shown in black color. (b) Analyses based on the evolution of backbone RMSDs of the bound (orange line) and unbound (blue line) systems indicated that the systems stabilized during the course of MD simulations. **Figure S2.** The 3D structures of small molecule-free PD-L1 dimers (in cartoon representation) showing the residues (stick representation) forming key H-bond interactions. The small molecule binding site (based on the small molecule bound PD-L1 dimer complex) is shown as a surface in white.

**Additional file 2.** Supplementary material

**Additional file 3.** Supplementary material

**Additional file 4.** Supplementary material

**Additional file 5.** Supplementary material

## Data Availability

The datasets supporting the conclusions of this article are included within the article and within the Additional files.

## Abbreviations

2D-QSAR: Two dimensional–quantitive structure activity relationship
hPD-L1: Human programmed death-ligand 1
PD-1: Programmed death-1
MAB: Monoclonal antibody
CTLA-4: Cytotoxic T lymphocyte–associated antigen 4
irAE: Immune-related adverse events
APCs: Antigen presenting cells
Tregs: Regulatory T cells
BMS: Bristol-Myers-Squibb
HTRF: Homogeneous time-resolved fluorescence
GIST: Grid Inhomogeneous Solvation Theory
HMQC: Heteronuclear Multiple-Quantum Correlation
NMR: Nuclear Magnetic Resonance
SD: Steepest descent
CG: Conjugated gradient
MM-GBSA: Molecular mechanics with generalized Born and surface area solvation
HSA: Hydration Site Analysis
MD: Molecular dynamics
RMS: Root mean square
FWA: Free Wilson analysis
CTLs: CD8.^+^ cytotoxic T lymphocytes
PPI: Protein–protein interaction
RMSD: Root mean square deviation
