# Supplementary material for: Leveraging structural and 2D-QSAR to investigate the role of functional group substitutions, conserved surface residues and desolvation in triggering the small molecule-induced dimerization of hPD-L1
Source: BMC Chem. 2022 Jun 27;16(1):49. doi: 10.1186/s13065-022-00842-w (PMC9238240; doi:10.1186/s13065-022-00842-w)
Supplement: Supplementary file 1 — Additional file 1: Figure S1. The 3D structure of a small molecule free PD-L1 dimer (a) and the comparison of backbone RMSD fluctuations in small molecule-bound and unbound systems during 75 ns long MD simulation (b). (a) In the 3D structure of the PD-L1 dimer shown as a cartoon representation, the chain A is shown in blue and chain B in Red. The region corresponding to the residues 33-42 and 93-105 in both the chains, where a physical restraint of 0.5 kcal/mol were applied, are shown in black color. (b) Analyses based on the evolution of backbone RMSDs of the bound (orange line) and unbound (blue line) systems indicated that the systems stabilized during the course of MD simulations. Figure S2. The 3D structures of small molecule-free PD-L1 dimers (in cartoon representation) showing the residues (stick representation) forming key H-bond interactions. The small molecule binding site (based on the small molecule bound PD-L1 dimer complex) is shown as a surface in white. [file 13065_2022_842_MOESM1_ESM.docx]

**Leveraging Structural, 2D-QSAR and Machine Learning Modeling to Investigate the Role of Functional Group Substitutions, Conserved Surface Residues and Desolvation in Triggering the Small Molecule-Induced Dimerization of hPD-L1**

Marawan Ahmed^1^, Aravindhan Ganesan^2^, and Khaled Barakat^1,3*^

^1^Faculty of Pharmacy and Pharmaceutical Sciences, University of Alberta, Edmonton, AB, Canada.

^2^ArGan's Lab, School of Pharmacy, University of Waterloo, Kitchener, ON, Canada.

^3^Li Ka Shing Institute of Virology, University of Alberta, Edmonton, Alberta, Canada.

***Corresponding Author: kbarakat@ualberta.ca**

**Figure S1: The 3D structure of a small molecule free PD-L1 dimer (a) and the comparison of backbone RMSD fluctuations in small molecule-bound and unbound systems during 75 ns long MD simulation (b)**. (a) In the 3D structure of the PD-L1 dimer shown as a cartoon representation, the chain A is shown in blue and chain B in Red. The region corresponding to the residues 33-42 and 93-105 in both the chains, where a physical restraint of 0.5 kcal/mol were applied, are shown in black color. (b) Analyses based on the evolution of backbone RMSDs of the bound (orange line) and unbound (blue line) systems indicated that the systems stabilized during the course of MD simulations.


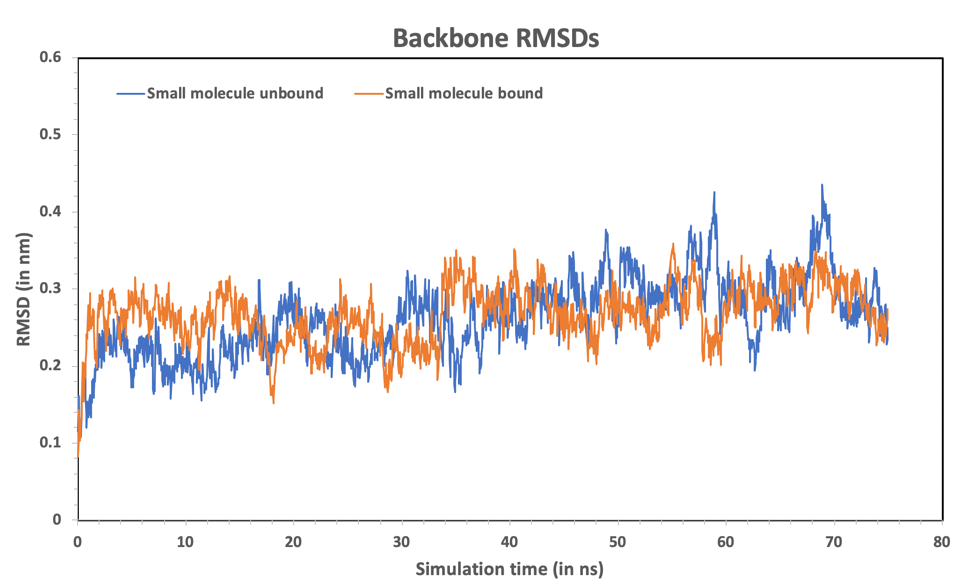

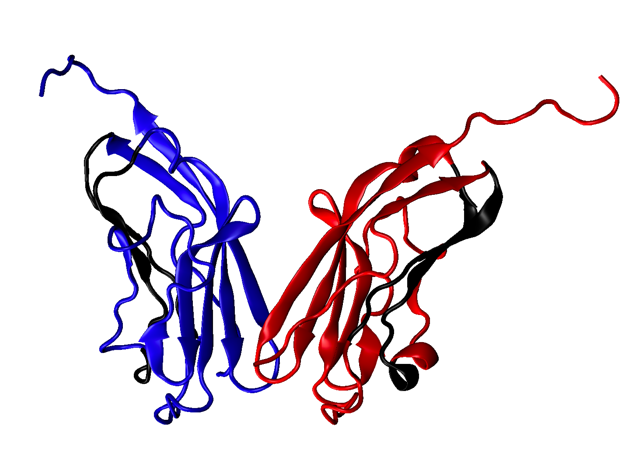


(b)

(a)

**Figure S2: The 3D structures of small molecule-free PD-L1 dimers (in cartoon representation) showing the residues (stick representation) forming key H-bond interactions.** The small molecule binding site (based on the small molecule bound PD-L1 dimer complex) is shown as a surface in white.


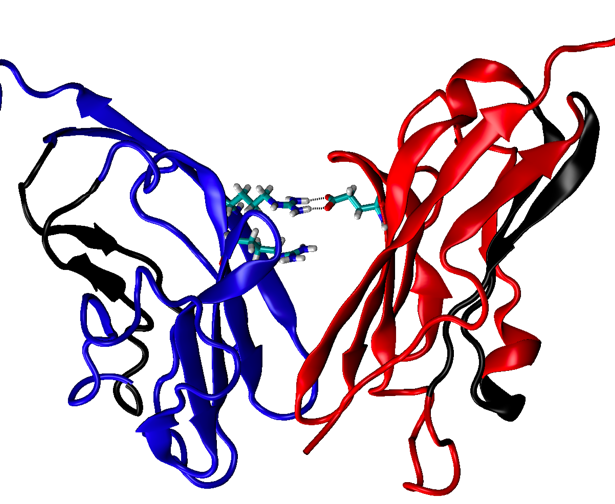


**R113**

**R126**

s

**E58**

**PD-L1 chain A**

s

**PD-L1 chain B**

s


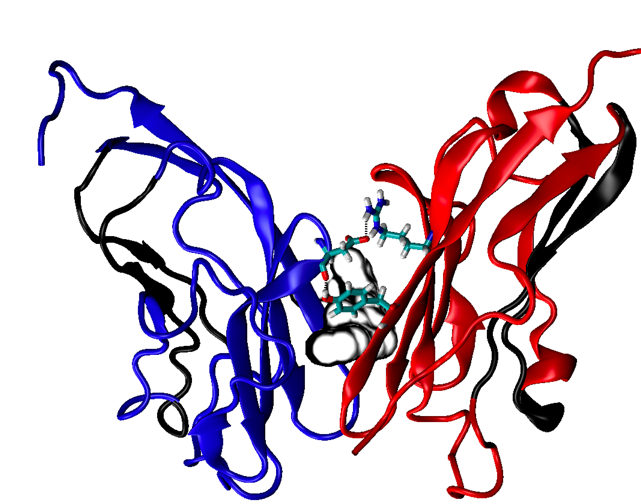


**R113**

s

**D61**

s

**Y123**

s

**PD-L1 chain A**

s

**PD-L1 chain B**

s


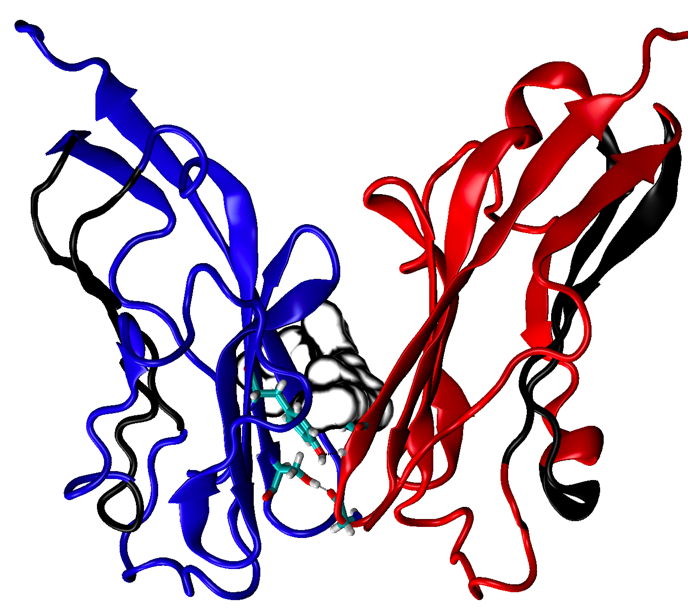


**S117**

**SG119**

s

***Y56-A121**

*****

s

**PD-L1 chain A**

s

**PD-L1 chain B**

s
